# Evaluation of mass mercury poisoning cases occurring in a center in Türkiye: symptomatology, treatment methods, and follow-up processes

**DOI:** 10.1007/s00431-025-06143-3

**Published:** 2025-04-23

**Authors:** Fatih Kurt, Abdullah Akcil, Sengul Cangur, Mustafa Yıldız

**Affiliations:** 1 Pediatrics Clinic, Duzce University, Duzce, Turkey; 2 Pediatrics Clinic, Sakarya University Training and Research Hospital, Adapazarı, Sakarya Turkey; 3 Biostatistics and Medical Informatics Department, Duzce University, Duzce, Turkey; 4 Pediatrics Clinic, Elazığ Fethi Sekin City Hospital, Elazığ, Turkey

**Keywords:** Mercury poisoning, DMPS (2,3-dimercaptopropane sulfonic acid), Elemental mercury, Urine mercury level, Blood mercury level

## Abstract

Mercury (Hg) is a toxic heavy metal with extensive applications. In children, mercury exposure often occurs inadvertently through laboratories, thermometers, or fluorescent lamps. Inhalation of elemental mercury can affect the central nervous system and urinary system. Early diagnosis and treatment are crucial to prevent severe complications. A retrospective evaluation was conducted on 82 pediatric cases of mercury poisoning who presented to Bingöl State Hospital on January 15, 2020. Blood and urine mercury levels were measured. Patients with mercury levels > 10 μg/L received intravenous treatment with 2,3-dimercaptopropane sulfonic acid (DMPS) for 5 days. Plasma and urine mercury levels were analyzed before and after treatment. Adverse effects of treatment and follow-up processes were also examined. Of the patients, 43.9% were female, and 56.1% were male, with a mean age of 9.4 ± 3.2 years. A total of 42.7% of cases were symptomatic, with headache being the most common symptom (26.8%). Significant reductions in blood and urine mercury levels were observed after treatment (*p* < 0.001). Adverse effects of the drug were reported in 43.9% of cases, with nausea (50%) and itching (25%) being the most frequent.

*Conclusion*: Chelation therapy was effective in significantly reducing mercury levels in cases of mercury poisoning. Adverse effects must be carefully managed, and long-term follow-up is essential. This study provides significant contributions to the literature on mass mercury poisoning cases.

**What is Known:**

• *Symptoms of mercury poisoning.*

**What is New:**

• *Long-term outcomes of DMPS therapy in 82 patients.*

## Introduction

Mercury (Hg) is a heavy metal with a wide range of applications and is frequently used. It exists in three different forms: organic, inorganic, and elemental. All of these forms are toxic; however, the most toxic form, which tends to bioaccumulate, is organic mercury, known as methylmercury. Elemental mercury exists as a liquid at room temperature. Mercury is a volatile substance, and inhalation of its vapor is the most common route of exposure. Even brief inhalation can lead to poisoning, as approximately 80% of inhaled mercury is absorbed by the lung parenchyma. Owing to its lipophilic properties, mercury is capable of crossing the blood‒brain and placental barriers [1]. Mercury is commonly used in household products, medicine, agriculture, and industry. In children, mercury exposure from broken thermometers, blood pressure devices, or fluorescent lamps is typically unintentional and may occur in chemical laboratories. The onset of symptoms varies depending on the form of mercury, its concentration, the route of exposure, and the duration of exposure [2].

Ingestion or dermal contact of elemental mercury generally does not produce toxic effects because of its low absorption in the gastrointestinal system and limited dermal penetration. However, the inhalation of elemental mercury can affect multiple systems, primarily the central nervous system and the urinary system. Early symptoms may be mild and nonspecific, making diagnosis challenging. The most commonly reported symptom is headache. Additionally, fatigue, abdominal pain, loss of appetite, gastrointestinal complaints, irritability, shyness, insomnia, and personality changes may occur. In some cases, a condition known as “acrodynia,” characterized by pink discoloration and peeling of the skin on the hands and feet, may be observed. Intense exposure to mercury vapor can lead to pneumonia in a short time and, subsequently, death due to progressive hypoxia [3].

Early diagnosis and treatment of mercury intoxication are crucial for preventing severe complications. Patients diagnosed with mercury poisoning should receive chelation therapy with a succimer, dimercaprol, or penicillamine [4].

In the incident discussed in our study, students took mercury from the chemistry laboratory without permission and removed it from the school premises. As a result, the exact quantity of mercury, the individuals who were exposed, the manner of exposure, and the duration of exposure could not be clearly determined. Therefore, the blood and 24-h urine mercury levels of all 360 individuals, including students, teachers, and those who were thought to have been exposed to mercury outside the school, were assessed. Chelation therapy with DMPS was initiated for the 82 patients whose blood or urine mercury levels exceeded 10 μg/L, whereas the other patients were monitored for symptoms. The symptoms, laboratory findings, chelation therapies administered, treatment-related side effects, and posttreatment follow-up processes of these 82 patients were retrospectively analyzed, with the aim of contributing to the literature.

## Materials and methodology

On January 15, 2020, 35 patients presented to the Pediatric Emergency Department of Bingöl State Hospital with symptoms including headache, abdominal pain, maculopapular rash, and nausea. Based on clinical findings, a history of mercury exposure, and blood and urine mercury level analyses, the patients were diagnosed with mercury poisoning. Following the incident, an epidemiological assessment was conducted to evaluate the extent of exposure. The next day, blood and urine mercury level tests were performed on a total of 325 individuals, including all students, teachers, and other individuals at risk of exposure in the affected school. Among the total of 360 cases subjected to laboratory analysis, it was determined that 82 cases, including those who presented to the emergency department and those in the at-risk group, had blood mercury levels exceeding 10 μg/L. These individuals were hospitalized, and intravenous DMPS treatment was initiated for 5 days, while those with lower blood mercury levels were advised to seek hospital care if symptoms developed. A total of 82 cases with blood mercury levels > 10 μg/L were included in the study.

The patients intentionally came into contact with mercury but without awareness of its toxic effects; some experienced exposure through both dermal contact and inhalation, whereas others were exposed solely via inhalation. Sociodemographic characteristics (e.g., age, sex), presenting complaints (e.g., headache, abdominal pain, joint pain, nausea, skin rash, pruritus), and medical history of the patients were recorded. Blood gas analysis, complete blood count (CBC), blood coagulation parameters, thyroid stimulating hormone (TSH), free T4 (fT4), plasma electrolytes, plasma mercury levels, and 24-h urinary mercury levels were evaluated, and electrocardiograms (ECGs) were performed for all patients. Patients with blood or 24-h urinary mercury levels > 10 μg/L were treated with intravenous 2,3-dimercaptopropane sulfonic acid (DMPS) chelation therapy for 5 days.

On the third day of treatment, follow-up assessments, including complete blood count (CBC), plasma electrolytes, fibrinogen, creatinine, aspartate aminotransferase (AST), alanine aminotransferase (ALT), creatine kinase (CK), international normalized ratio (INR), thyroid function tests, and urinalysis, were performed. Following intravenous DMPS therapy, plasma and 24-h urinary mercury levels were re-evaluated. Patients with persistent mercury levels > 10 μg/L in blood or 24-h urine samples were transitioned to oral DMPS therapy and subsequently monitored.

For all patients, complete blood count (CBC), plasma electrolytes, fibrinogen, creatinine, aspartate aminotransferase (AST), alanine aminotransferase (ALT), thyroid function tests, and urinalysis were evaluated after treatment, at the 1-week mark, and at the 1-month mark. All patients were scheduled for follow-up at the 6-month mark, during which plasma and 24-h urinary mercury levels, and other control tests were planned. However, none of the patients attended their 6-month follow-up appointments. The blood mercury levels of all students attending the school where the incident occurred and all other cases exposed to mercury were measured. Patients with blood mercury levels < 10 μg/L were not given chelation therapy and were excluded from the study.

In our study, the clinical findings, plasma, and 24-h urinary mercury levels, complete blood count, plasma electrolyte levels, TSH and free T4 levels, the number of administered treatment doses, and treatment-related adverse effects in 82 cases undergoing chelation therapy were retrospectively examined. Additionally, among these 82 cases with blood mercury levels > 10 μg/L, the blood and urinary mercury levels of symptomatic and asymptomatic individuals were compared.

### Statistical analysis

The quantitative variables in the study are presented as the means ± standard deviations and medians [interquartile ranges (IQRs)], whereas the qualitative data are expressed as frequencies and percentages. The normality of the quantitative variables was assessed via the Shapiro‒Wilk test. The Mann‒Whitney *U* test was used for comparisons between groups. For comparisons of variables measured at different time points, the Friedman two-way ANOVA (post hoc: Dunn test) and the Wilcoxon signed-rank test were used. A nonparametric partial correlation coefficient was calculated to assess the relationships between quantitative variables by controlling for confounding factors. Statistical analyses were performed via SPSS version 22 software, and a *p* value of < 0.05 was considered to indicate statistical significance.

## Results

A total of 82 children participated in the study; 43.9% were girls, and 56.1% were boys. The mean age was 9.4 ± 3.2 years. In 12.2% of the cases, exposure occurred through both dermal and inhalation routes, whereas the remaining cases were exposed solely via inhalation. Headache was observed in 26.8% of the children, maculopapular rash in 15.9%, nausea in 9.8%, and abdominal pain in 8.5%. Among the patients, 42.7% were symptomatic, whereas 57.3% were asymptomatic. The proportion of patients with abnormal ECG findings at the initial examination was 12.2%, and among the 10 patients with abnormal ECG results, 90% had right bundle branch block, whereas 10% had T-wave negativity. The proportion of patients with drug side effects was 43.9%. Among the 36 patients with side effects, 50% had nausea, 25% had itching, 19.4% had arm pain, 16.7% had abdominal pain and fatigue, and 13.9% experienced headache and thrombophlebitis. The sociodemographic-clinical characteristics of the patients and the drug side effects recorded in the patients are given in detail in Table 1 and Table 2, respectively.

**Table 1:** Sociodemographic characteristics, symptoms and clinical findings of the patients (*n* = 82)

		***n***	%
Gender	Girl	36	43.9
	Boy	46	56.1
Age, years*		9.4 ± 3.2 (1–14)	
Type of exposure	Skin and inhalation	10	12.2
	Inhalation	72	87.8
Comorbidities		3	3.7
**Presence of symptoms**		35	42.7
Headache		22	26.8
Dizziness		1	1.2
Eye pain		2	2.4
Abdominal pain		7	8.5
Nausea		8	9.8
Maculopapular rash		13	15.9
Itching		4	4.9
Joint pain		2	2.4
**Clinical findings**			
Abnormal ECG		10	12.2
Cases with abnormal ECG	Incomplete right branch block	9	90
	Negative T wave	1	10

*Mean ± standard deviation (minimum–maximum)

**Table 2:** Drug side effects reported in the patients

		***n***	**%**
Presence of drug side effects		36	43.9
Drug side effects (*n* = 36)	Nausea	18	50
	Itching	9	25
	Arm pain	7	19.4
	Abdominal pain	6	16.7
	Fatigue	6	16.7
	Headache	5	13.9
	Thrombophlebitis	5	13.9
	Rash	2	5.6
	Vomiting	1	2.8

The mercury levels measured in the blood and 24-h urine samples of the patients at different time points are presented in Table 3. A statistically significant difference was observed between blood mercury levels measured at admission and after 5 days of intravenous DMPS therapy, with a significant reduction in blood mercury levels on day 5 (*p* < 0.001) (Table 3). A statistically significant difference was also found among the blood mercury levels measured at different periods in the 17 patients who received oral treatment (*p* < 0.001). Compared with other measurements, the blood mercury level at admission was the highest, whereas the blood mercury level after oral treatment was the lowest (*p* < 0.05) (Table 3).

**Table 3:** Mercury levels measured in blood and urine at different time points

	Mean **± **SD	Median [IQR]	***p***	***p*′**	***p*″**
Blood mercury level at admission (*n* = 82)	46.13 ± 55.10	23.82 [31.37]	** < 0.001**	** < 0.001**	**0.011**^**#**^ ** < 0.001**^**$**^ **0.011**^**£**^
Blood mercury level in fifth day (*n* = 82)	8.88 ± 16.01	2.88 [6.49]			
Blood mercury level after oral treatment (*n* = 17)	4.03 ± 2.49	4.48 [3.4]			
Urine mercury level within 24-h (*n* = 82)	27.95 ± 52.17	5.54 [13.57]	**0.004**	** < 0.001**	**0.049**^**#**^ ** < 0.001**^**$**^ **0.011**^**£**^
Urine mercury level in fifth day (*n* = 82)	13.11 ± 21.76	4.87 [8.51]			
Urine mercury level after oral treatment (*n* = 17)	5.00 ± 3.43	4.31 [5.27]			

*SD* standard deviation, *IQR* interquartile range

*p* value for comparison between admission and the fifth day

*p* value for comparison among admission, fifth day, and after oral treatment

*p* value for post hoc tests

#: *p* value for comparison of admission vs. fifth day

$: *p* value for comparison of admission vs. after oral treatment

£: *p* value for comparison of the fifth day vs. after oral treatment

Similarly, a statistically significant difference was observed between 24-h urinary mercury levels measured at admission and on day 5, with a significant decrease in urinary mercury levels on day 5 (*p* < 0.05, Table 3). Significant differences were also found in 24-h urinary mercury levels measured at different periods in the 17 patients who received oral treatment (*p* < 0.001). Compared with other measurements, the urinary mercury level at admission was the highest, whereas the level after oral treatment was the lowest (*p* < 0.05) (Table 3).

The distributions and graphs of cases showing increases or decreases in biochemical parameters relative to normal reference values at different time points are presented in Table 4, respectively. Before treatment, the highest rates of increase or decrease compared with normal values were observed for the international normalized ratio (INR): INR (11%), total bilirubin (2.4%), and aspartate transaminase (AST) (2.4%). By the third day of treatment, although the rate of INR increase had decreased (6.1%), increases in the rates of fibrinogen reduction (3.7%), plasma ALT (6.1%), AST (4.9%), creatinine (3.7%), CK elevation (2.4%), leukocyte (WBC) count (1.2%), and eosinophil count (1.2%) were observed.

**Table 4:** Distribution of cases showing increases or decreases in biochemical parameters compared with normal values at different time points (*n* = 82)

	***n***	%
**Before intravenous DMPS therapy**		
INR increase (+)	9	11
Total bilirubin increase (+)	2	2.4
Fibrinogen decrease (+)	1	1.2
ALT increase (+)	1	1.2
AST increase (+)	2	2.4
Creatinine increase (+)	1	1.2
**Day 3 of treatment**		
INR increase (+)	5	6.1
Total bilirubin increase (+)	1	1.2
Fibrinogen decrease (+)	3	3.7
ALT increase (+)	5	6.1
AST increase (+)	4	4.9
Creatinine increase (+)	3	3.7
CK increase (+)	2	2.4
Increase in WBC count (+)	1	1.2
Increase in eosinophil count (+)	1	1.2
**After treatment**		
INR increase (+)	1	1.2
Total bilirubin increase (+)	1	1.2
Fibrinogen decrease (+)	2	2.4
Increase in WBC count (+)	1	2.4
Increase in eosinophil count (+)	1	1.2
**One week after treatment**		
ALT increase (+)	2	2.4
AST increase (+)	2	2.4
Increase in WBC count (+)	1	1.2
Increase in eosinophil count (+)	1	1.2
Plasma TSH change (+)	4	4.9
Proteinuria (+)	1	1.2
**One month after treatment**		
INR increase (+)	4	4.9
Increase in WBC count (+)	1	1.2
Increase in eosinophil count (+)	1	1.2
Proteinuria (+)	4	4.9

*INR* international normalized ratio, *AST* aspartate aminotransferase, *ALT* alanine aminotransferase, *CK* creatine kinase, *WBC* white blood cell, *TSH* thyroid-stimulating hormone

Following intravenous DMPS therapy, reductions in fibrinogen levels and increases in leukocyte counts were identified in 2.4% of the 82 patients. One week after treatment, alterations in TSH levels were detected in four patients (4.9%). In two patients, plasma TSH levels were below the lower limit of the reference range, whereas in the other two patients, they exceeded the upper limit. Additionally, elevated AST and ALT levels were recorded in two patients (2.4%). One month after treatment, INR elevation was identified in four patients (4.9%), and proteinuria was detected in four patients.

The data on mercury levels measured in blood and 24-h urine samples on the basis of the presence of symptoms are presented in Table 5. A statistically significant difference was observed in mercury levels in blood and urine based on the presence of symptoms (*p* < 0.05) (Table 5). The median blood mercury level in symptomatic patients was 42.79 [IQR = 82.81], whereas in asymptomatic patients, it was 20.08 [IQR = 17.19]. The median urinary mercury level in symptomatic patients was 16.5 [IQR = 111.84], whereas in asymptomatic patients, it was 3.96 [IQR = 4.04]. Additionally, when examining the relationship between mercury levels in blood and urine, there were no significant confounding effects of sex or the presence of comorbidities (*p* > 0.05), except for age (*p* < 0.05). In this case, a positive and statistically significant partial correlation was found between mercury levels in blood and urine after controlling for age (*r* = 0.524, *p* < 0.001).

**Table 5:** Mercury levels measured in blood and 24-h urine samples on the basis of the presence of symptoms

	Presence of symptom				
	Present (***n*** = 35)		Absent (***n*** = 47)		***p***
	Mean ± SD	Median [IQR]	Mean ± SD	Median [IQR]	
**Blood mercury level**	73.25 ± 73.41	42.79 [82.81]	25.94 ± 19.68	20.08 [17.19]	**0.001**
**Urine mercury level**	57.99 ± 69.36	16.5 [111.84]	5.58 ± 6.21	3.96 [4.04]	** < 0.001**

*SD* standard deviation, *IQR* interquartile range

## Discussion

Mercury is the only liquid metal on Earth and has become a global pollutant, posing a significant threat to human health and the ecological system. The toxicity of mercury is determined by its form, duration of exposure, and route of exposure. Even small amounts of mercury can vaporize in enclosed spaces, leading to symptoms [1]. In our study, a total of 82 cases were examined, involving individuals who had come into contact with elemental mercury—obtained without authorization from a school laboratory—either at school or at home, who exhibited symptoms or were found to have elevated urine and plasma mercury levels.

Studies have reported a correlation between blood or urine mercury levels and the severity of toxicity, but symptoms can be observed even when urine mercury levels are less than 10 μg/L [5]. In our study, when symptomatic and asymptomatic patients were compared, the mercury levels in the blood and urine of the symptomatic patients were significantly greater. None of our symptomatic patients had blood or urine mercury levels less than 10 μg/L. Halbach and colleagues examined the blood and urine mercury concentrations in individuals with amalgam fillings and reported that both blood and urine mercury levels decreased similarly after the removal of the amalgam fillings [6]. In our study, a significant positive relationship was observed between the blood and urine mercury concentrations of the patients after controlling for age (*r* = 0.524, *p* < 0.001).

Not all cases of mercury poisoning present with symptoms. A study conducted by Güven et al. reported that 46.9% of cases of mercury poisoning were asymptomatic [2]. In our study, 57.4% of the patients were asymptomatic. When mercury poisoning is symptomatic, the symptoms are often nonspecific and multisystemic, which can complicate the diagnosis and lead to confusion with rheumatological diseases, endocrine disorders, and infectious diseases [1, 7, 8]. Studies have reported that the most common symptom is headache; in the study by Carman et al., headache was reported in approximately 24% of the cases [9]. In our cases, headache was also the most common symptom, present in 26.8% of the cases.

Studies have reported that cardiac parameters such as heart rate, rhythm, contractility, and excitability are affected by mercury intoxication [10]. In our study, we evaluated the electrocardiograms (ECGs) of all patients and found abnormalities in 12.2% of them. Among these abnormalities, 90% were incomplete right bundle branch blocks, and in one 4-year-old patient, we observed T wave negativity in leads V1, V2, and V3. The literature suggests that incomplete right bundle branch blocks, which are more common in athletes, can be found in 10–20% of the general population [11]. Since no cardiac pathology developed during the follow-up of our patients, we concluded that these electrocardiographic changes were not related to mercury intoxication.

In the treatment of mercury poisoning, the primary step is to remove the source of exposure. In cases of skin contact, the affected area should be washed with soap and water, and if the eyes are involved, they should be rinsed with saline solution. The patient’s airway, respiratory, and circulatory systems should be evaluated, and respiratory and circulatory support should be provided if necessary. Chelation therapy agents such as Dimercaprol (BAL), dimercaptosuccinic acid (DMSA), D-penicillamine (DPCN), and DMPS are commonly used [12]. The oral absorption of DMPS is 39%, and it is more stable than DMSA when administered intravenously, with a half-life of 20 h. However, it has been reported to be ineffective in removing methylmercury from the brain. In our study, DMPS therapy was administered to the patients. Initially, intravenous DMPS was given for 5 days, followed by 5 days of oral DMPS if necessary. Treatment was completed for patients whose mercury levels in both blood and 24-h urine returned to normal. Twelve of our patients received oral DMPS once, three received it twice, and two received it five times. When the mercury levels in the blood and 24-h urine at the time of presentation, after intravenous therapy, and after oral therapy were compared, a significant reduction in mercury levels was observed following both treatments. These findings demonstrate the beneficial effects of both intravenous and oral DMPS therapy in our patients.

DMPS is a sulfone-containing preparation with a low incidence of side effects. The expected side effects include rash, hypersensitivity reactions, nausea, and leukopenia [13]. In our study, side effects were observed in 43.9% of the patients. The most common side effects were nausea, which was observed in 50% of the patients with side effects, and pruritus, which was observed in 25% of the patients. Only two of our patients developed morbiliform skin rashes, and leukopenia was not observed in any of the patients. In addition to these side effects, extremely rare cases have been reported in the literature. Owing to its sulfone content, DMPS may be associated with the development of Stevens–Johnson syndrome (SJS). A case of SJS related to the DMPS has been documented in the literature [14]. Additionally, a case of fixed drug eruption induced by DMPS has also been reported [15]. However, these rare adverse effects were not observed in the patients included in our study. Studies have reported cases of thrombophlebitis associated with chelators such as CaNa2 EDTA [16]. However, we did not find any reported cases of thrombophlebitis related to the DMPS in the literature. In our study, thrombophlebitis developed in 5 of our patients during intravenous DMPS chelation therapy.

Symptoms such as pruritus, nausea, and headache can occur due to both mercury poisoning and DMPS treatment. If these symptoms were present at the time of hospital admission, they were attributed to mercury poisoning. However, if they developed after the initiation of DMPS treatment in the absence of these symptoms at admission, they were considered adverse effects of the DMPS.

Bellinger et al. reported that mercury can accumulate in kidney tissues, leading to proteinuria, nephrotic syndrome, and even kidney failure [17]. In our study, none of the patients presented with proteinuria in early urine samples. However, one patient developed proteinuria of 150 mg/dL in the first week after treatment, and the proteinuria levels returned to normal during the first month of follow-up. During the first-month follow-up, 4 patients had proteinuria levels between 30 and 100 mg/dL, and in all 4 of these patients, proteinuria decreased over time. These findings suggest that mercury may accumulate in kidney tissues and cause proteinuria in the later stages of exposure.

Studies have reported that mercury intoxication can lead to renal failure, hepatic failure, multiorgan failure, and disseminated intravascular coagulation (DIC) [18, 19]. In our study, prior to treatment, all patients underwent hemogram, AST, ALT, creatinine, creatine kinase, INR, prothrombin time (PT), activated partial thromboplastin time (aPTT), total bilirubin, direct bilirubin, and fibrinogen tests. During the follow-up, control tests were conducted on the 3rd day of treatment, after intravenous therapy, 1 week after treatment, and 1 month after treatment. The laboratory results revealed that the most frequent abnormality was an elevated international normalized ratio (INR), which was found in 9 patients. Mild increases in AST were observed in five patients, ALT in four patients, creatinine in three patients, and creatine kinase (CK) in two patients. Additionally, hypofibrinogenemia developed in three patients. The clinical condition of the patients was monitored, and no deterioration in their overall health was observed during follow-up. The results of the control tests revealed that the values returned to normal within one month. No cases of hepatic failure, renal failure, or DIC were observed. Studies have also reported that eosinophilia and leukocytosis can occur during mercury poisoning [20]. In our study, one patient had a WBC count of 33,000, with 57% of the white blood cells being eosinophils. The peripheral smear of this patient showed normal cell morphology. After other potential causes were excluded, these findings were thought to be due to mercury intoxication. The patient’s WBC elevation and eosinophilia persisted at the first-month follow-up, but 2 months later, these values had returned to normal.

A study conducted by Hu et al. reported that mercury has an endocrine-disrupting effect on thyroid hormone homeostasis, and as blood mercury levels increase, plasma TSH and free T4 levels also increase [21]. Soldin et al. reported that methylmercury interferes with TSH production, thereby disrupting thyroid function [22]. Numerous studies in the literature have demonstrated that mercury can affect thyroid function in various ways. In our study, two patients presented elevated TSH levels, whereas two other patients presented low TSH levels. Concurrent free T4 levels remained within normal limits. The plasma TSH levels in these four patients normalized within 3 weeks.

Studies have demonstrated that the long-term effects of mercury exposure may persist, including during the intrauterine period. In a study conducted by Yüksel et al., a significant correlation was identified between maternal and fetal cord blood mercury levels [23]. Another study reported that elevated mercury levels in maternal and neonatal blood may be associated with intrauterine growth restriction [24]. Additionally, other research has suggested that prenatal mercury exposure could lead to permanent impairments in neurodevelopmental and cognitive functions in the long term. Postnatal mercury exposure has been suggested to contribute to pulmonary dysfunction, potentially leading to childhood asthma and chronic obstructive pulmonary disease (COPD) in adulthood. Furthermore, mercury has been reported to be associated with cerebellar ataxia, nystagmus, and mild cognitive impairments. Hepatic dysfunction and alterations in gut microbiota may result in gastrointestinal dysfunction, whereas immune suppression can increase susceptibility to infections, autoimmune diseases, and systemic inflammation. In the cardiovascular system, endothelial dysfunction, thrombosis, and cardiomyopathy have been reported, while in the renal system, asymptomatic functional loss may progress to significant dysfunction over time. Ophthalmologically, a reduction in the retinal nerve fiber layer and choroidal thickness has been observed [25–28]. At the first-month follow-up, no clinical symptoms or findings related to the long-term effects of mercury exposure were observed in the study participants. However, since the participants did not attend the 6-month follow-up, it was not possible to assess whether any symptoms had emerged in the later period.

## Conclusion

This study provides comprehensive data on the symptomatology, treatment processes, and follow-up outcomes of mass poisoning cases caused by elemental mercury exposure. Chelation therapy was found to significantly reduce mercury levels and achieve clinical improvement; however, the necessity of carefully managing side effects during the treatment process was emphasized. These findings highlight the importance of early diagnosis, effective treatment, and long-term follow-up in cases of mercury poisoning. With 82 cases, this study offers significant contributions to the limited literature on mass mercury poisoning incidents. Furthermore, this study evaluated the efficacy of both intravenous and oral chelation therapies, providing valuable clinical insights into treatment approaches.

This study emphasizes the need for safe laboratory practices and the restriction of mercury-containing materials in schools, as well as the necessity for training teachers and students to recognize the symptoms of mercury poisoning.

## Limitations

Symptoms such as headache, nausea, and itching can occur both as mercury intoxication and as adverse effects of DMPS. If these symptoms were present at the time of admission, we attributed them to mercury intoxication; if they appeared after the initiation of DMPS, we considered them adverse effects of the drug. Additionally, the exaggeration of symptoms caused by mass hysteria may have influenced the frequency of symptoms. This study’s retrospective design inherently limits the data collection process to existing records, with no possibility of obtaining additional information. Since the study focuses solely on cases from a single center, the generalizability of the findings is restricted. Furthermore, the lack of long-term follow-up data prevents a comprehensive evaluation. Additionally, the exaggeration of symptoms by the patients due to mass hysteria may have influenced the frequency of symptoms of potential late complications associated with mercury exposure.

## Data Availability

No datasets were generated or analysed during the current study.

## Abbreviations

DMPS: 2,3-Dimercaptopropane sulfonic acid
INR: International normalized ratio
AST: Aspartate aminotransferase
ALT: Alanine aminotransferase
CK: Creatine kinase
WBC: White blood cell
TSH: Thyroid-stimulating hormone
